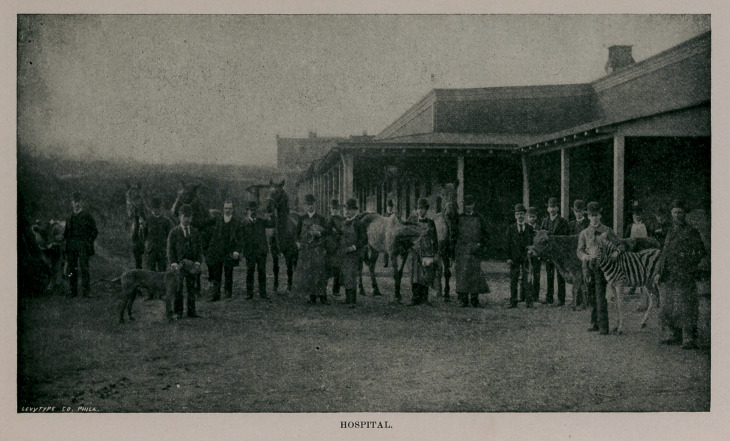# Veterinary Department—University of Pennsylvania

**Published:** 1888-07

**Authors:** William Osler


### Art. XX.—THE VETERINARY DEPARTMENT OF THE UNIVERSITY OF PENNSYLVANIA.

The authorities of the University of Pennsylvania undertook an ambitious experiment when, four years ago, they founded the Veterinary Department. The plan was deliberately laid some time before, and as the proper person to undertake it did not appear to be available, Dr. Rush Shippen Huidekoper, one of the Demonstrators of Surgery in the Medical Faculty, a man of recognized energy and capacity, was asked to go abroad and in the best European schools qualify himself for the task. On his return, in 1883, the scheme was matured, and in October, 1884, the school was opened with twenty-nine matriculates.

The intention from the outset was to furnish an educa-

VETERINARY DEPARTMENT, UNIVERSITY OF PENNSYLVANIA.

tion in veterinary medicine on advanced lines, and the curriculum of study was modelled on the basis of the French and German schools, with a preliminary examination and a graded course of studies extending over three years, with sessions of nine months' duration. During the first year instruction was given only in the elementary branches, and it was not until the session of 1885-86 that the infirmary buildings were completed. The course of study is most extensive and thorough, and, with the exception of Harvard, longer than in any medical school in the country. In the first year the subjects studied are Chemistry, Materia Medica and Pharmacy, Histology, Botany, Zoölogy, Veterinary Anatomy and Forging. A very special advantage in this session is the courses which the students take in Botany and Zoölogy at the Biological Department. I feel convinced, from my Montreal experience, that this is a most beneficial plan for veterinary students, as it not only extends their knowledge, but tends greatly to heighten their appreciation of the scientific aspects of the profession.

In the second year the subjects taught are Medical Chemistry, Physiology, Therapeutics, General Pathology and Morbid Anatomy, Veterinary Anatomy, Zoölogy, Surgical Pathology and Internal Pathology, and the Contagious Diseases. The General Pathology and Chemistry are taken at the Medical School, where the students have the advantage of the extensive chemical laboratories.

In the third year the subjects of the curriculum are: Therapeutics, General Pathology and Morbid Anatomy, Surgical Pathology and Operative Surgery, Internal Pathology and the Contagious Diseases, Sanitary Police, Obstetrics and Zoötechnics. In the second year the student attends clinics, while in the third year he is placed in charge of sick animals, prepares the clinical reports and makes autopsies.

The teaching facilities of this school are exceptionally good, equal to many of the European, and superior to most veterinary colleges of this country. The explanation is simple—more money has been spent. The situation has been most fortunately chosen, and when the projected botanical garden is arranged, the place will rival in beauty of surroundings the Berlin school. The Infirmary stables are commodious and well arranged, and there is a separate department for irrigation and baths. The dog kennels are large and roomy, and a distant portion of the building has been assigned for an experimental station. One of the special features of the school is the farriery—a cut of which is given—in which the student has to learn practically all the details of normal and pathological shoeing. The dissecting room, the histological, physiological and pharmaceutical laboratories are arranged on the most modern plans, and by the exertion of Dr. Huidekoper the Museum is annually enriched by valuable specimens, particularly in comparative osteology.

After an existence of four sessions, we should be able to form some idea of the prospects of the school. Naturally, with strict requirements and a prolonged course, a large number of students could not be expected; but in the session of 1886-87 there were forty-nine in attendance, and the first graduating class contained eleven men. In the session just completed the number was fifty-six, and there were thirteen graduates. The chief difficulty in maintaining a high grade school is the heavy expense entailed in the costly laboratory and hospital equipments. The income derived from the students, necessarily limited in number, is insufficient for maintenance, and endowment is absolutely necessary. The State may be looked to for material assistance, as the very existence of such a school in our midst is of the greatest possible benefit to the agriculturist; but in the future, as in the past, it is to generous citizens, like the late Mr. J. B. Lippincott, that the school must look for endowments. The practical success so far attained has been due to the unceasing exertion of Dr. Huidekoper, who has devoted himself early and late to the interests of the school. The University has indeed been fortunate in securing a man to organize the department who has high aims, a proper conception of the dignity of his profession, and, above all, a due appreciation of the fact that education in any line of life, to be worthy of the name, must be thorough.

## Figures and Tables

**Figure f1:**
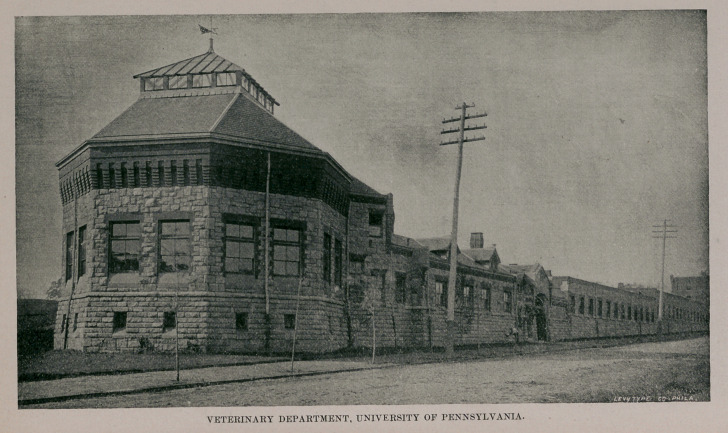


**Figure f2:**
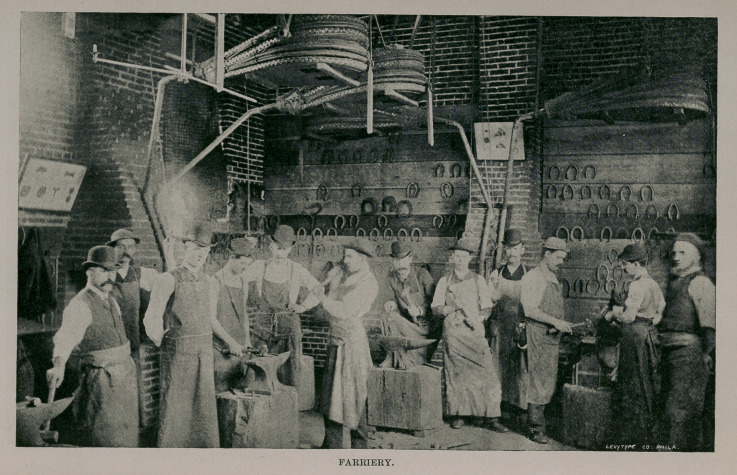


**Figure f3:**